# Low Prognostic Nutritional Index (PNI) Predicts Unfavorable Distant Metastasis-Free Survival in Nasopharyngeal Carcinoma: A Propensity Score-Matched Analysis

**DOI:** 10.1371/journal.pone.0158853

**Published:** 2016-07-11

**Authors:** Lin Yang, Liangping Xia, Yan Wang, Shaodong Hong, Haiyang Chen, Shaobo Liang, Peijian Peng, Yong Chen

**Affiliations:** 1 Sun Yat-sen University cancer center, Guangzhou, China; 2 State Key Laboratory of Oncology in Southern China, Guangzhou, China; 3 Collaborative Innovation Center for Cancer Medicine, Guangzhou, China; 4 The Sixth Affiliated Hospital of Sun Yat-sen University, Guangzhou, China; 5 The First Hospital of Foshan, Foshan, China; 6 The Fifth Affiliated Hospital of Sun Yat-sen University, Zhuhai, China; Van Andel Institute, UNITED STATES

## Abstract

**Background:**

Poor nutritional status is associated with progression and advanced disease in patients with cancer. The prognostic nutritional index (PNI) may represent a simple method of assessing host immunonutritional status. This study was designed to investigate the prognostic value of the PNI for distant metastasis-free survival (DMFS) in patients with nasopharyngeal carcinoma (NPC).

**Methods:**

A training cohort of 1,168 patients with non-metastatic NPC from two institutions was retrospectively analyzed. The optimal PNI cutoff value for DMFS was identified using the online tool “Cutoff Finder”. DMFS was analyzed using stratified and adjusted analysis. Propensity score-matched analysis was performed to balance baseline characteristics between the high and low PNI groups. Subsequently, the prognostic value of the PNI for DMFS was validated in an external validation cohort of 756 patients with NPC. The area under the receiver operating characteristics curve (AUC) was calculated to compare the discriminatory ability of different prognostic scores.

**Results:**

The optimal PNI cutoff value was determined to be 51. Low PNI was significantly associated with poorer DMFS than high PNI in univariate analysis (P<0.001) as well as multivariate analysis (P<0.001) before propensity score matching. In subgroup analyses, PNI could also stratify different risks of distant metastases. Propensity score-matched analyses confirmed the prognostic value of PNI, excluding other interpretations and selection bias. In the external validation cohort, patients with high PNI also had significantly lower risk of distant metastases than those with low PNI (Hazards Ratios, 0.487; P<0.001). The PNI consistently showed a higher AUC value at 1-year (0.780), 3-year (0.793) and 5-year (0.812) in comparison with other prognostic scores.

**Conclusion:**

PNI, an inexpensive and easily assessable inflammatory index, could aid clinicians in developing individualized treatment and follow-up strategies for patients with non-metastatic NPC.

## Introduction

Nasopharyngeal carcinoma (NPC) has a distinct ethnical and geographical distribution and is one of the most common types of head and neck cancer. The annual incidence of NPC in some areas of southern China and South East Asia is 20–30 cases per 100,000 [1, 2]. Approximately 80% of patients present with advanced disease at first diagnosis as a result of its silent, deep-seated location and non-specific symptoms[3]. Its association with the Epstein–Barr virus (EBV), higher radio- and chemo-sensitivity and greater propensity for distant metastasis differentiate NPC from non-nasopharyngeal head and neck squamous cell carcinomas [4]. The advent of intensity-modulated radiotherapy and the development of more precise imaging technologies improved loco-regional control, and the risk of distant metastasis has been reduced by the application of systemic chemotherapy [5]. However, more than 20% of patients with advanced disease still develop distant metastasis after radical radiotherapy and the survival outcomes remain unsatisfactory, even after salvage treatment. Therefore, distant metastasis has become the major pattern of treatment failure in NPC, making accurate prognostic evaluation at diagnosis extremely important for optimal therapy [5, 6].

The current staging TNM classification system remains the most widely used prognostic tool for stratification and the design of therapeutic strategies for patients with NPC[7]. However, the TNM classification focuses solely on tumor behavior, regardless of the host response. Unsurprisingly, heterogeneous survival outcomes are observed for patients with equivalent TNM classifications [8, 9]. Therefore, identification of an accurate prognostic indicator is warranted to supplement the TNM classification and recognize patients at high risk of distant metastasis.

Epstein-Barr virus (EBV), a high intake of salt-preserved foods and genetic factors has been confirmed to contribute to the development of cancer [10–12]. However, there is increasing evidence that host-related factors, malnutrition and cancer-related inflammation may also promote tumor development, progression and metastasis by damaging the immune system and altering tumor cell biology within the tumor microenvironment [11, 13, 14]. Hypoalbuminemia is often observed in patients with advanced cancer and is regarded as an index of malnutrition and cachexia. Hypoalbuminemia is associated with a reduced quality of life, treatment toxicity, poor response to treatment and shorter survival, and is an independent predictor of poor survival in several types of cancer including gastrointestinal cancer, lung cancer, ovarian cancer and breast cancer, as well as NPC [15–18].

Assessment of the systemic immunonutritional status has been refined by introduction of the prognostic nutritional index (PNI), a continuous variable based on serum albumin concentration and total lymphocyte count in peripheral blood. The PNI was originally designed to assess perioperative immunonutritional status and surgical risk in patients undergoing gastrointestinal surgery [17]. Additionally, the PNI can also indicate systemic inflammation, which has been associated with tumorigenesis and cancer progression [19]. Recently, the prognostic value of the PNI has been validated in a variety of malignant tumor types, including colorectal cancer, gastric cancer, malignant pleural mesothelioma, hepatocellular carcinoma and pancreatic cancer [20–22]. However, the application of mean or median values determined in patients with other types of cancer as cutoff points is arbitrary and may not be useful when assessing the true prognostic value of a variable. Moreover, no studies have investigated the prognostic role of PNI in non-metastatic NPC.

We hypothesized that immunonutritional status assessed by PNI is associated with distant metastasis-free survival (DMFS) in patients with NPC. Therefore, the aim of this propensity score-matched analysis was to evaluate whether PNI has prognostic value for DMFS in patients with NPC after adjusting for potential confounding factors.

## Materials and Methods

### Training cohort

A total of 1,168 eligible patients with NPC treated at Sun Yat-sen University Cancer Center or the First Hospital of Foshan between October 2007 and December 2009 were retrospectively enrolled using the same inclusion criteria, which were: (i) patients with pathological evidence of NPC; (ii) with complete baseline clinical information and laboratory data; (iii) who received radical radiotherapy (iv) and with complete follow-up data. Patients with distant metastasis at presentation were excluded. Ethical approval was obtained from both institutions through the respective institutional review boards. The requirement for informed consent was waived due to the retrospective nature of the study. The study protocol was designed in accordance with the guidelines outlined in the Declaration of Helsinki and was approved by the Ethics Committee of Sun Yat-sen University Cancer Center and the First Hospital of Foshan.

A standardized data collection form was designed to retrieve all relevant sociodemographic data (age, gender, smoking history); baseline laboratory data (EBV DNA copy number), albumin (ALB), alanine transaminase (ALT), aspartate transaminase (AST), lactate dehydrogenase (LDH), alkaline phosphatase (ALP), C-reactive protein (CRP), etc; staging data (T stage, based on the location, size and extension of the primary tumor; N stage, based on the number and location of lymph node metastases); and therapeutic data (radiotherapy technique, chemotherapy). Clinical stage was reclassified according to the seventh edition of the AJCC/UICC TNM Staging System.

### Validation cohort

To examine the predictive accuracy of the PNI, an external validation cohort of 756 consecutive patients with NPC from the Fifth Affiliated Hospital of Sun Yat-sen University between January 2007 and December 2010 were retrospectively enrolled under the same criteria. Only patients with non-metastatic disease were included; sufficient data to assess the prognostic value of the PNI was available for all patients.

### Follow up

Distant metastasis was evaluated by physical examinations, nasopharyngoscope, nasopharyngeal and neck magnetic resonance imaging (MRI), chest x-ray and/or CT scans, abdominal ultrasonography and bone scans every three months during the first three years after the completion of radiotherapy and annually thereafter. DMFS was defined as the time from the complete response of definitive radiotherapy to the time of metastases or censorship at the date of last follow-up.

### Statistical analysis

Continuous variables were expressed as mean ± standard deviation, median and range, and were transformed into dichotomous variables at median value. Comparisons were performed using the chi-square test or Fisher’s exact test for categorical variables and Mann-Whitney test for continuous variables.

The PNI was calculated as 10 × serum albumin value (g/dl) + 0.005 × peripheral lymphocyte count (per mm^3^)[23]. The optimal cutoff level for the neutrophil to lymphocyte ratio (NLR), platelet to lymphocyte ratio (PLR) and PNI was determined using the web-based system Cutoff Finder designed by Budczies J et al. (http://molpath.charite.de/cutoff/) [24]. The modified Glasgow Prognostic Score (mGPS) was entered into the analysis as categorical variables as descried before [25]

DMFS curves were plotted using the Kaplan–Meier method, with comparisons between groups performed using the log-rank test. Cox regression models were used to assess the relationships between the PNI and DMFS; with adjustment for variables with significant level of <0.2 in the univariate analysis and/or that were clinically expected to be of importance. Results are expressed as hazards ratios (HRs) with 95% percent confidence intervals. Subgroups were defined using significant factors in univariate analysis or factors that correlated directly with the PNI in the chi-square test; namely, age, gender, smoking status, ALT, AST, ALP, LDH, WBC (White cell), HGB (Hemoglobin),CRP, EBV-DNA level, radiotherapy technique, treatment method, T category, N category and clinical stage.

To overcome biases due to the different distributions of co-variables among the groups of patients with a low PNI and high PNI, propensity score-match (PSM) analysis was performed using R software version 2.9.0 (R Project for Statistical Computing, Vienna, Austria) via one-to-two matching and using a small caliper of 0.15 to ensure even distributions. A number of 548 patients for whom the propensity score could not be matched were excluded from further analysis. The following co -variables were matched: age, gender, smoking status, WBC, neutrophil count, HGB, ALT, AST, ALP, LDH, CRP, EBV-DNA level, radiotherapy technology, T category, N category, treatment method. Finally, the index was subjected to external validation using the Kaplan–Meier method to verify the prognostic value of the PNI. Statistical analyses of survival data were performed using SPSS 19.0 for Windows (SPSS, Chicago, IL, USA). Two-sided *P* values < 0.05 were deemed significant.

## Results

### Patient characteristics and survival

A total of 1168 and 756 patients from the training dataset and the external validation dataset, respectively, were included. Median follow-up for DMFS was 68.8 months in the training dataset and 60.25 months in the validation dataset. Five-year DMFS rate was 85.6% in the training dataset and 83.5% in the validation dataset. The baseline characteristics of the included patients are provided in Table 1.

**Table 1:** Clinical and laboratory characteristics of the patients in the training set and validation set.

Characteristic	Training set	Validation set
	Number of cases (%)	Number of cases (%)
**Age, years**		
< 45	602 (51.5)	403 (53.3)
≥ 45	566 (48.5)	353 (46.7)
**Gender**		
Male	853 (73)	556 (73.5)
Female	315 (27)	200 (26.5)
**Smoking status**		
Absent	705 (60.4)	498 (65.9)
Present	463 (39.6)	258 (34.1)
**WBC, ×10**^**9**^**/L**		
< 6.9	608 (52.1)	413 (54.6)
≥ 6.9	560 (47.9)	343 (45.4)
**Neutrophils, ×10**^**9**^**/L**		
< 4.1	601 (51.5)	400 (52.9)
≥ 4.1	567 (48.5)	356 (47.1)
**HGB, g/L**		
< 143	596 (51)	369 (48.8)
≥ 143	572 (49)	387 (51.2)
**ALT, U/L**		
< 20.6	585 (50.1)	358 (47.4)
≥ 20.6	583 (49.9)	398 (52.6)
**AST, U/L**		
< 20.8	588 (50.3)	377 (49.9)
≥ 20.8	580 (49.7)	379 (50.1)
**ALP, U/L**		
< 66.7	591 (50.6)	373 (49.3)
≥ 66.7	577 (49.4)	383 (50.7)
**LDH, U/L**		
< 166	583 (49.9)	379 (50.1)
≥ 166	585 (50.1)	377 (49.9)
**EBV-DNA, copies/ml**		
< 1,000	500 (42.8)	382 (50.5)
1,000–9,999	230 (19.7)	144 (19.1)
10,000–99,999	281 (24.1)	152 (20.1)
> 100,000	157 (13.4)	78 (10.3)
**CRP, mg/L**		
< 1.49	583 (49.9)	382 (50.5)
≥ 1.49	585 (50.1)	374 (49.5)
**ALB, g/L**		
< 45.6	585 (50.6)	350 (46.3)
≥ 45.6	583 (49.9)	406 (53.7)
**Radiotherapy technique**		
CRT	496 (42.5)	498 (65.9)
IMRT + 3DCRT	672 (57.5)	258 (34.1)
**Treatment method**		
Radiotherapy	220 (18.8)	182 (24.1)
CCRT	494 (42.3)	244 (32.2)
Neo + radiotherapy	210 (18.0)	152 (20.1)
Neo + CCRT	244 (20.9)	178 (23.5)
**T category**		
1	76 (6.5)	69 (9.1)
2	300 (25.7)	171 (22.6)
3	547 (46.8)	358 (47.4)
4	245 (21.0)	158 (20.9)
**N category**		
0	246 (21.1)	162 (21.4)
1	425 (36.4)	305 (40.3)
2	310 (26.5)	201 (26.6)
3	187 (16.0)	88 (11.6)
**Clinical stage**		
I	29 (2.5)	28 (3.7)
II	199 (17)	120 (15.9)
III	620 (53.1)	413 (54.6)
IV	320 (27.4)	195 (25.8)
**Distant metastasis**		
Absent	981 (84.0)	627 (82.9)
Present	187 (16.0)	129 (17.1)
**Survival status**		
Live	952 (81.5)	632 (83.6)
Dead	216 (18.5)	124 (16.4)

Abbreviations: WBC, white blood cells; HGB, hemoglobin; ALT, alanine transaminase; AST, aspartate transaminase; ALP, alkaline phosphatase; LDH, lactate dehydrogenase; CRP, C-reactive protein; ALB, albumin; PNI, prognostic nutritional index; EBV-DNA, Epstein-Barr virus DNA; DMFS, distant metastasis-free survival; RT, radiotherapy; CCRT, concurrent radiotherapy; Neo, neoadjuvant chemotherapy; CRT, conventional radiotherapy: 3D-CRT, three-dimensional conformal radiotherapy; IMRT, intensity-modulated radiation therapy;

### Prognostic value of the PNI for DMFS in the training cohort and external cohort undergoing definitive radiotherapy

**Fig 1:**
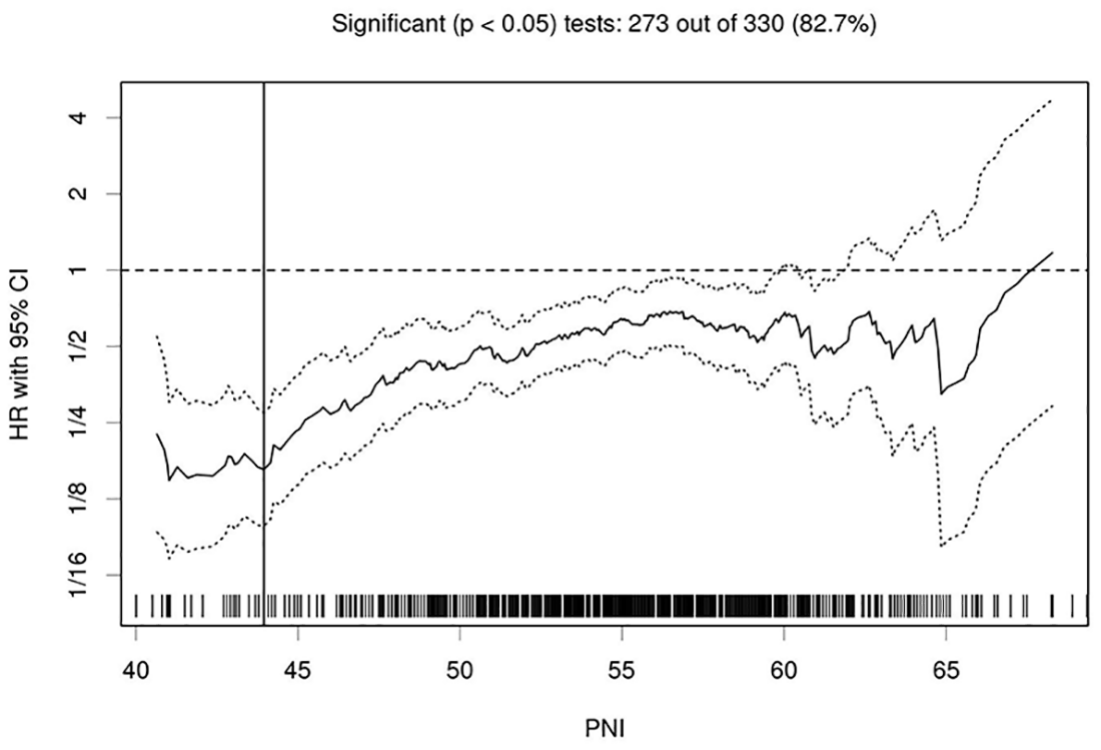
Hazard ratio (HR) for distant metastasis-free survival (DMFS) independent of the cutoff point for prognostic nutritional index (PNI) in patients with nasopharyngeal carcinoma. The vertical line designates the optimal cutoff points with the most significant split (log-rank test). The plots were generated using Cutoff Finder.

The median value of PNI in the training and external cohorts were 55.18 and 54.32, respectively. Using the Cutoff Finder tool, we determined the optimal cutoff for PNI with respect to DMFS to be 51; this value provided the greatest separation of the DMFS curves in Kaplan-Meier analysis (Fig 1). PNI was significantly associated with age, gender, WBC, HGB, ALT, CRP and N classification in the training cohort by the chi-square test (Table 2).

**Table 2:** Associations between PNI and clinicopathological features before and after propensity score matching.

Characteristic	PNI (before matching)			PNI (after matching)		
	Low	High	P	Low	High	P
**Age, years**			0.002			0.953
< 45	78 (14.1%)	474 (85.9%)		78 (33.5%)	155 (66.5%)	
≥ 45	129 (20.9%)	487 (79.1%)		129 (33.2%)	259 (66.8%)	
**Gender**			0.009			1.000
Male	136 (15.9%)	717 (84.1%)		136 (33.3%)	272 (66.7%)	
Female`	71 (22.5%)	244 (77.5%)		71 (33.3%)	142 (66.7%)	
**Smoking status**			0.761			0.954
Absent	123 (17.4%)	582 (82.6%)		123 (33.4%)	245 (66.6%)	
Present	84 (18.1%)	379 (81.9%)		84 (33.2%)	169 (66.8%)	
**WBC, ×10**^**9**^**/L**			0.042			0.646
< 6.9	121 (19.9%)	487 (80.1%)		121 (34.1%)	234 (65.9%)	
≥ 6.9	86 (18.1%)	474 (84.6%)		86 (32.3%)	180 (67.7%)	
**Neutrophils, ×10**^**9**^**/L**			0.817			0.210
< 4.1	105 (17.5%)	496 (82.5%)		105 (31.2%)	232 (68.8%)	
≥ 4.1	102 (18.0%)	465 (82.0%)		102 (35.9%)	182 (64.1%)	
**HGB, g/L**			<0.001			0.812
< 143	135 (22.7%)	461 (77.3%)		135 (33.7%)	266 (66.3%)	
≥ 143	72 (12.6%)	500 (87.4%)		72 (32.7%)	148 (67.3%)	
**ALT, U/L**			0.002			0.490
< 20.6	124 (21.2%)	461 (78.8%)		124 (34.4%)	236 (65.6%)	
≥ 20.6	83 (14.2%)	500 (85.8)		83 (31.8%)	178 (68.2%)	
**AST, U/L**			0.375			0.955
< 20.8	110 (18.7%	478 (81.3%)		110 (33.4%)	219 (66.6%)	
≥ 20.8	97 (16.7%)	483 (83.3%)		97 (33.2%)	195 (66.8%)	
**ALP, U/L**			0.100			0.460
< 66.7	94 (15.9%)	497 (84.1%)		94 (31.9%)	201 (68.1%)	
≥ 66.7	113 (19.6%)	464 (80.4%)		113 (34.7%)	213 (65.3)	
**LDH, U/L**			0.573			0.820
< 166	107 (18.4%)	476 (81.6%)		107 (33.8%)	210 (66.2%)	
≥ 166	100 (17.1%)	485 (82.9%))		100 (32.9%)	204 (67.1%)	
**CRP, mg/L**			<0.001			0.560
< 1.49	77 (13.2%)	506 (86.8%)		77 (32.0%)	164 (68.0%)	
≥ 1.49	130 (22.2%)	455 (77.8%)		130 (34.2%)	250 (65.8%)	
**EBV-DNA, copies/ml**			0.196			0.987
< 1,000	76 (15.2%)	424 (84.8%)		76 (33.3%)	152 (66.7%)	
1,000–9,999	41 (17.8%)	189 (82.2%)		41 (32.8%)	84 (67.2%)	
10,000–99,999	59 (21.0%)	222 (79.0%)		59 (34.3%)	113 (65.7%)	
> 100,000	31 (19.7%)	126 (80.3%)		31 (32.3%)	65 (67.7%)	
**Radiotherapy technique**			0.284			1.000
CRT	81 (16.3%)	415 (83.7%)		81 (33.3%)	162 (66.7%)	
IMRT + 3DCRT	126 (18.8%)	546 (81.3%)		126 (33.3%)	252 (66.7%)	
**Treatment method**			0.116			0.692
Radiotherapy	34 (15.5%)	186 (84.5%)		34 (35.4%)	62 (64.6%)	
CCRT	81 (16.4%)	413 (83.6%)		81 (31.9%)	173 (68.1%)	
Neo radiotherapy	49 (23.3%)	161 (76.7%)		49 (36.8%)	84 (63.2%)	
Neo + CCRT	43 (17.6%)	20 (82.4%)		43 (31.2%)	95 (68.8%)	
**T category**			0.189			0.721
1	9 (11.8%)	67 (88.2%)		9 (36.0%)	16 (64.0%)	
2	49 (16.3%)	251 (83.7%)		49 (36.0%)	87 (64.0%)	
3	96 (17.6%)	451 (82.4%)		96 (31.2%)	212 (68.8%)	
4	53 (21.6%)	192 (78.4%)		53 (34.9%)	99 (65.1%)	
**N category**			0.034			0.951
0	36 (14.6%)	210 (85.4%)		36 (35.0%)	67 (65.0%)	
1	64 (15.1%)	361 (84.9%)		64 (32.2%)	135 (67.8%)	
2	66 (21.3%)	244 (78.7%)		66 (34.2%)	127 (65.8%)	
3	41 (21.9%)	146 (78.1%)		41 (32.5%)	85 (67.5%)	

Abbreviations: WBC, white blood cells; HGB, hemoglobin; ALT, alanine transaminase; AST, aspartate transaminase; ALP, alkaline phosphatase; LDH, lactate dehydrogenase; CRP, C-reactive protein; ALB, albumin; PNI, prognostic nutritional index; EBV-DNA, Epstein-Barr virus DNA; DMFS, distant metastasis-free survival; RT, radiotherapy; CCRT, concurrent radiotherapy; Neo, neoadjuvant chemotherapy; CRT, conventional radiotherapy: 3D-CRT, three-dimensional conformal radiotherapy; IMRT, intensity-modulated radiation therapy;

**Fig 2:**
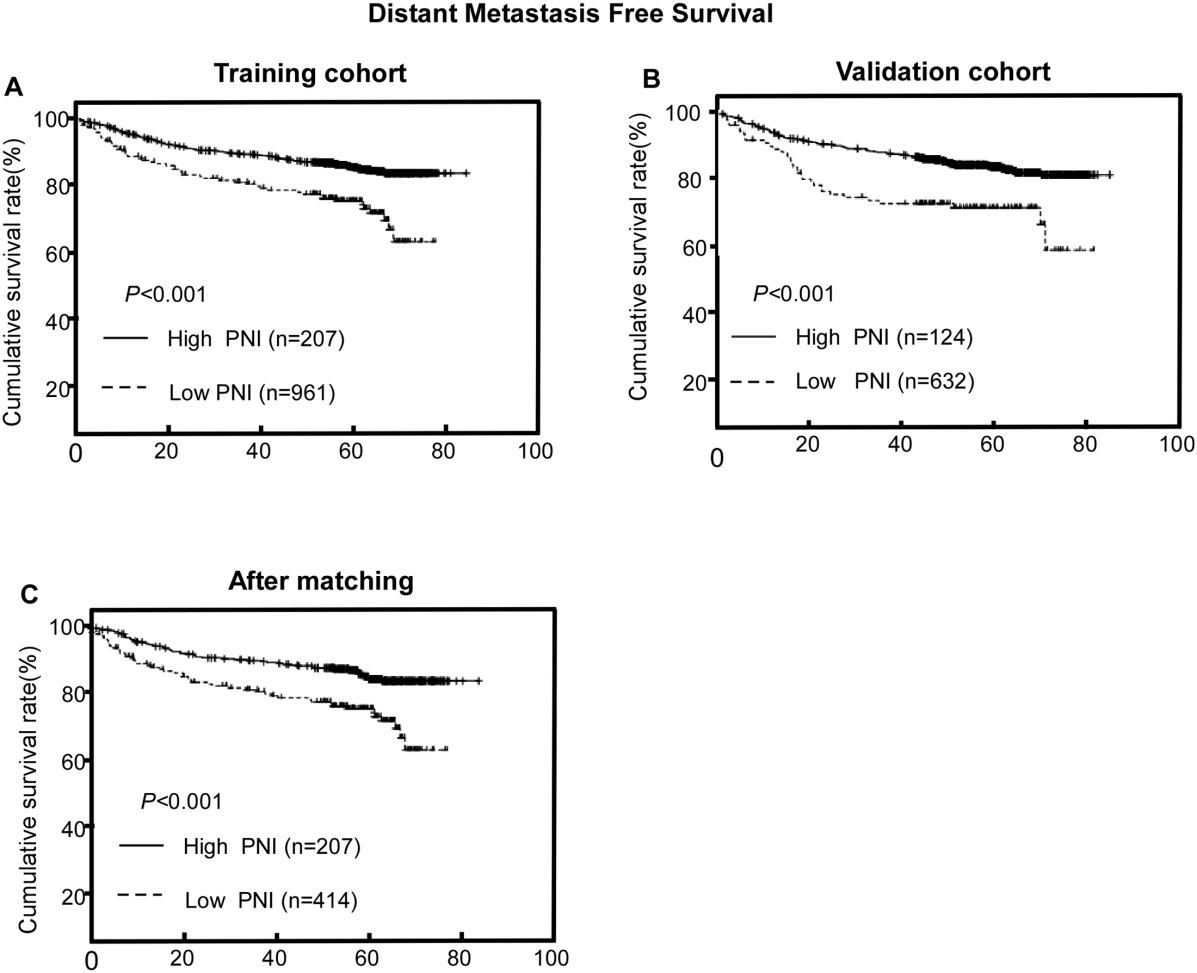
Prognostic value of the prognostic nutritional index (PNI) for distant metastasis-free survival (DMFS). (A) In the training cohort before matching, (B) the validation cohort and (C) the training cohort after 2:1 ratio matching.

**Fig 3:**
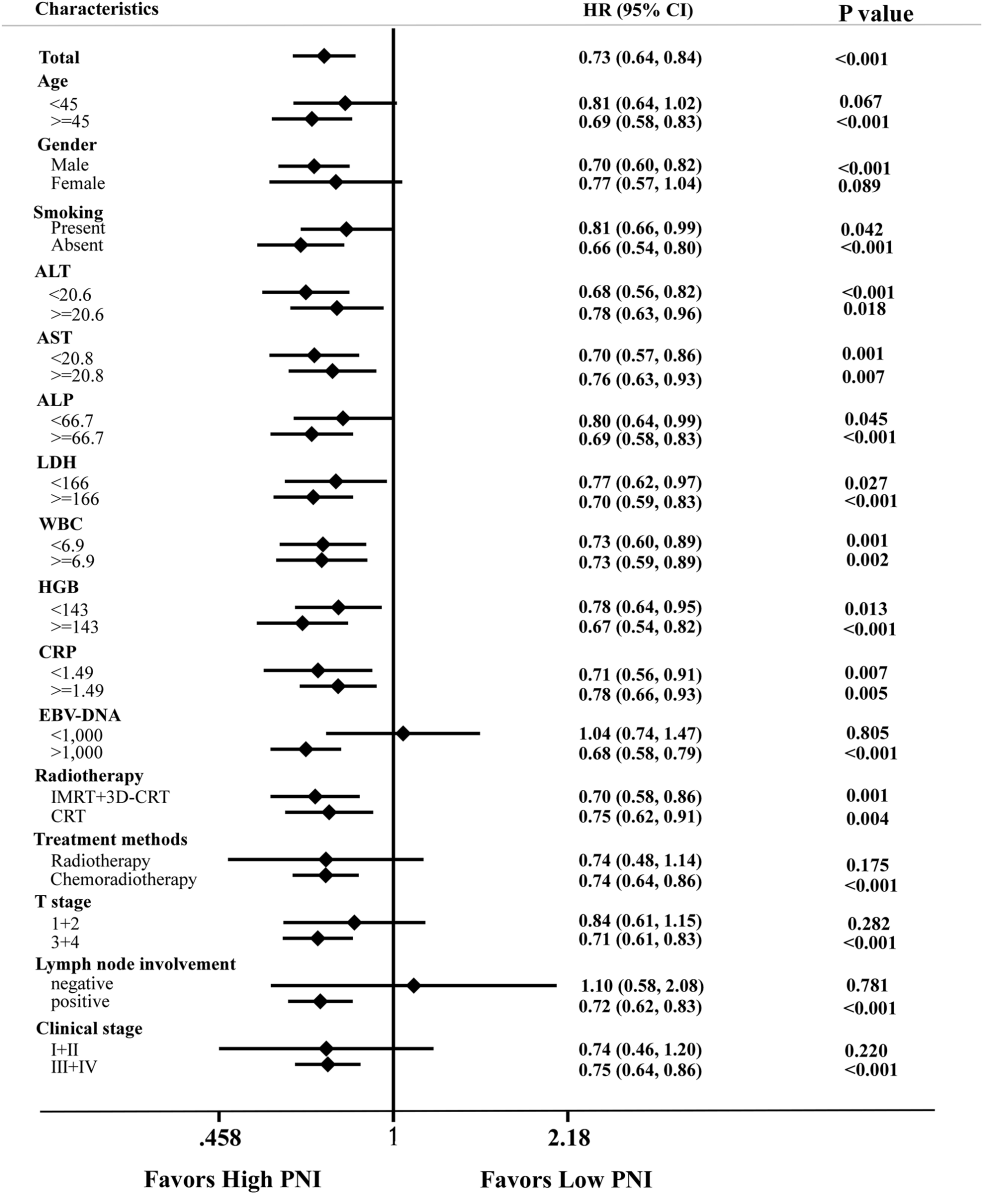
Forest plot of subgroup effects for distant metastasis-free survival (DMFS) in 1,168 patients with nasopharyngeal carcinoma who underwent definitive radiotherapy. Subgroups are defined by factors showing significant associations between the PNI and DMFS. Univariate hazard ratios and 95% CI (bars) are presented. WBC, white blood cell count; HGB, hemoglobin; ALT, alanine transaminase; AST, aspartate transaminase; ALP, alkaline phosphatase; LDH, lactate dehydrogenase; CRP, C-reactive protein; ALB, albumin; EBV, Epstein-Barr virus DNA; CRT, conventional radiotherapy: IMRT, intensity-modulated radiation therapy; 3D-CRT, three-dimensional conformal radiation therapy; RT, radiotherapy; chemo-radiotherapy, chemotherapy plus radiotherapy.

PNI had the ability to distinguish patients who developed distant metastasis in the training cohort by log-rank test (P < 0.001); this association was also validated in the external cohort (*P* < 0.001; Fig 2). After adjusting for potential confounding factors by multivariate analysis, the groups of patients with high and low PNI still had significantly different DMFS rates (HR = 0.419, P = 0.001; Table 3). Gender (P = 0.024), LDH (P = 0.017), CRP (P = 0.022), EBV-DNA copy number (P = 0.002), T category (P = 0.019), N category (P < 0.001), radiotherapy technique (P = 0.010) and PLR (P = 0.001) were also confirmed as independent prognostic factors for DMFS in the training cohort (Table 3). In addition, the prognostic value of PNI was consistent across different subgroups, with no identification of any interference (Fig 3).

**Table 3:** Univariable and multivariate analysis of associations with DMFS before propensity score matching.

Characteristic	Uni.			Multi.		
	HR	95%CI	*P*	HR	95% CI	*P*
**Age, years** (≥ 45 VS <45)	1.138	0.853–1.519	0.379			
**Gender** (Female VS Male)	0.673	0.473–0.958	0.028	0.660	0.460–0.947	0.024
**S Smoking status** (Present VS Absent)	1.484	1.113–1.979	0.007			
**WBC, × 10**^**9**^**/L** (≥ 6.9 VS <6.9)	1.180	0.885–1.573	0.260			
**HGB, g/L** (≥ 143 VS <143)	1.076	0.807–1.434	0.617			
**ALT, U/L** (≥ 20.6 VS <20.6)	1.260	0.944–1.683	0.117			
**AST, U/L** (≥ 20.8 VS <20.8)	1.237	0.927–1.651	0.148			
**ALP, U/L** (≥66.7 VS <66.7)	1.148	0.861–1.530	0.348			
**LDH, U/L** (≥ 166 VS <166)	1.712	1.275–2.300	<0.001	1.446	1.069–1.955	0.017
**CRP, mg/L** (≥ 1.49 VS <1.49)	1.875	1.392–2.525	<0.001	1.436	1.054–1.955	0.022
**EBV-DNA, copies/ml**			<0.001			0.002
< 1,000	1.000			1.000		
1,000–9,999	1.190	0.764–1.854	0.443	0.938	0.599–1.469	0.779
10,000–99,999	1.799	1.238–2.615	0.002	1.197	0.811–1.767	0.366
> 100,000	2.843	1.924–4.200	<0.001	1.997	1.336–2.984	0.001
**T category**			0.003			0.019
1	1.000			1.000		
2	0.745	0.378–1.471	0.397	0.725	0.364–1.443	0.360
3	1.053	0.562–1.973	0.872	0.860	0.455–1.628	0.644
4	1.635	0.856–3.125	0.137	1.362	0.706–2.629	0.357
**N category**			<0.001			<0.001
0	1.000			1.000		
1	1.750	1.024–2.992	0.041	1.591	0.928–2.727	0.091
2	2.681	1.574–4.565	<0.001	2.396	1.390–4.128	0.002
3	6.203	3.658–10.518	<0.001	5.088	2.972–8.712	<0.001
**Treatment method**			0.020			
Radiotherapy	1.000					
CCRT	0.554	0.327–0.937	0.028			
Neo + radiotherapy	1.009	0.693–1.470	0.961			
Neo + CCRT	1.272	0.829–1.951	0.271			
**Radiotherapy technique** (IMRT+3DCRT VS CRT)	0.715	0.536–0.953	0.022	0.682	0.510–0.911	0.010
**NLR(**≥1.105 VS <1.105**)**	2.238	0.992–5.049	0.052			
**PLR(**≥193.6 VS <193.6**)**	2.203	1.165–4.166	0.015	3.169	1.643–6.110	0.001
**mGPS**			0.005			
0	1.000					
1	1.843	1.190–2.853	0.006			
2	3.726	0.923–15.039	0.065			
**PNI** (≥51 VS <51)	0.490	0.355–0.677	<0.001	0.419	0.299–0.586	<0.001
*PNI			<0.001			
<42.1	1.000					
42.1–50.0	0.271	0.133–0.550	<0.001			
50.0–64.8	0.143	0.075–0.272	<0.001			
>64.8	0.027	0.003–0.208	0.001			
*PNI (continuous)	0.926	0.904–0.949	<0.001			

Abbreviations: WBC, white blood cells; HGB, hemoglobin; ALT, alanine transaminase; AST, aspartate transaminase; ALP, alkaline phosphatase; LDH, lactate dehydrogenase; CRP, C-reactive protein; EBV-DNA, Epstein-Barr virus DNA; DMFS, distant-metastasis free survival; RT, radiotherapy; CCRT, concurrent radiotherapy; Neo, neoadjuvant chemotherapy; CRT, conventional radiotherapy: 3D-CRT, three-dimensional conformal radiotherapy; IMRT, intensity-modulated radiation therapy; Uni., univariable; Multi., multivariate; PNI, prognostic nutritional index; NLR, the neutrophil to lymphocyte ratio; PLR, the platelet to lymphocyte ratio; mGPS, modified Glasgow Prognostic Score.

* Not entered into multivariate Cox-regression analysis.

Furthermore, when the PNI was analyzed as a continuous variable, the significance of DMFS discrimination by PNI still exists (HR = 0.926, P<0.001). That is to say, the metastatic risk of NPC patients decreases by 30% per 5 units’ increase in PNI. Also, when PNI was graded into four groups, the difference among DMFS in these four groups was still statistically significant (Table 3 and S1A Fig).

### Propensity score-matched analysis

Propensity score matching yielded a total of 621 patients from the training cohort (207 in high PNI and 414 in low PNI groups). After propensity matching, the distribution of confounding variables was remarkably balanced between the high and low PNI groups (Table 2).

PNI also had the ability to distinguish patients who developed distant metastasis after propensity matching by log-rank test (P < 0.001, Fig 2C); Univariate analysis revealed that patients with high PNI had lower probability of distant metastasis than those with low PNI (HR = 0.490, P < 0.001). (Table 4).Multivariate analyses revealed that PNI remained an independent prognostic factor for DMFS (P = 0.001). Compared to patients with low PNI (< 51), those with high PNI had an estimated 53% reduction in the risk of distant metastasis (HR, 0.458; 95% CI, 0.328–0.639; P <0.001) (Table 4).

**Table 4:** Univariable and multivariate analysis of associations with DMFS after 1:2 ratio propensity score matching.

Characteristic	Uni.			Multi.		
	HR	95%CI	*P*	HR	95% CI	*P*
**Age** (≥ 45 years VS <45)	1.138	0.854–1.889	0.238			
**Gender** (Female VS male)	0.551	0.355–0.856	0.008			
**S Smoking status** (Present VS Absent)	1.889	1.295–2.753	0.001	1.844	1.258–2.701	0.002
**WBC, × 10**^**9**^**/L** (≥ 6.9 VS <6.9)	1.183	0.811–1.724	0.383			
**HGB, g/L** (≥ 143 VS <143)	1.197	0.816–1.755	0.358			
**ALT, U/L** (≥ 20.6 VS <20.6)	1.267	0.870–1.846	0.217			
**AST, U/L** (≥ 20.8 VS <20.8)	1.273	0.874–1.854	0.208			
**ALP, U/L** (≥66.7 VS <66.7)	1.148	0.792–1.687	0.452			
**LDH, U/L** (≥ 166 VS <166)	1.673	1.141–2.453	0.008	1.588	1.077–2.342	0.020
**CRP, mg/L** (≥ 1.49 VS <1.49)	1.803	1.189–2.734	0.006			
**EBV-DNA, copies/ml**			<0.001			0.006
< 1,000	1.000					
1,000–9,999	1.135	0.595–2.163	0.701	1.094	0.573–2.089	0.786
10,000–99,999	2.332	1.406–3.868	0.001	1.845	1.100–3.096	0.020
> 100,000	3.112	1.818–5.326	<0.001	2.431	1.397–4.228	0.002
**T category**			0.057			
1	1.000					
2	3.863	0.520–28.723	0.187			
3	4.151	0.574–30.042	0.159			
4	6.433	0.882–46.921	0.066			
**N category**			<0.001			<0.001
0	1.000					
1	2.304	1.012–5.246	0.047	2.082	0.911–4.761	0.082
2	2.796	1.239–6.308	0.013	2.195	0.961–5.016	0.062
3	6.061	2.701–13.602	<0.001	4.953	2.176–11.274	<0.001
**Treatment method**			0.133			
Radiotherapy	1.000					
CCRT	1.221	0.653–2.283	0.532			
Neo + radiotherapy	1.918	1.006–3.654	0.048			
Neo + CCRT	1.262	0.642–2.478	0.500			
**Radiotherapy technique** (IMRT+3DCRT VS CRT)	0.868	0.593–1.269	0.464			
**NLR(**≥1.105 VS <1.105**)**	1.406	0.954–2.072	0.085			
**PLR(**≥193.6 VS <193.6**)**	1.518	1.116–2.065	0.008	2.192	1.276–3.764	0.004
**mGPS**			0.017			
0	1.000					
1	1.702	1.097–2.641	0.018			
2	3.300	0.816–13.340	0.094			
**PNI** (≥51 V<51)	0.490	0.355–0.677	<0.001	0.458	0.328–0.639	<0.001
*PNI			<0.001			
<42.1	1.000					
42.1–50.0	0.296	0.145–0.602	0.001			
50.0–64.8	0.156	0.082–0.298	<0.001			
>64.8	0.029	0.004–0.227	0.001			
*PNI (continuous)	0.930	0.906–0.955	<0.001			

Abbreviations: WBC, white blood cells; HGB, hemoglobin; ALT, alanine transaminase;AST, aspartate transaminase; ALP, alkaline phosphatase; LDH, lactate dehydrogenase; CRP, C-reactive protein; EBV-DNA, Epstein-Barr virus DNA; DMFS, distant-metastasis free survival; RT, radiotherapy; CCRT, concurrent radiotherapy; Neo, neoadjuvant chemotherapy; CRT, conventional radiotherapy: 3D-CRT, three-dimensional conformal radiotherapy; IMRT, intensity-modulated radiation therapy; Uni., univariable; Multi., multivariate; PNI, prognostic nutritional index; NLR, the neutrophil to lymphocyte ratio; PLR, the platelet to lymphocyte ratio; mGPS, modified Glasgow Prognostic Score.

* Not entered into multivariate Cox-regression analysis.

### Comparison of several inflammation-based prognostic scoring systems for DMFS

**Fig 4:**
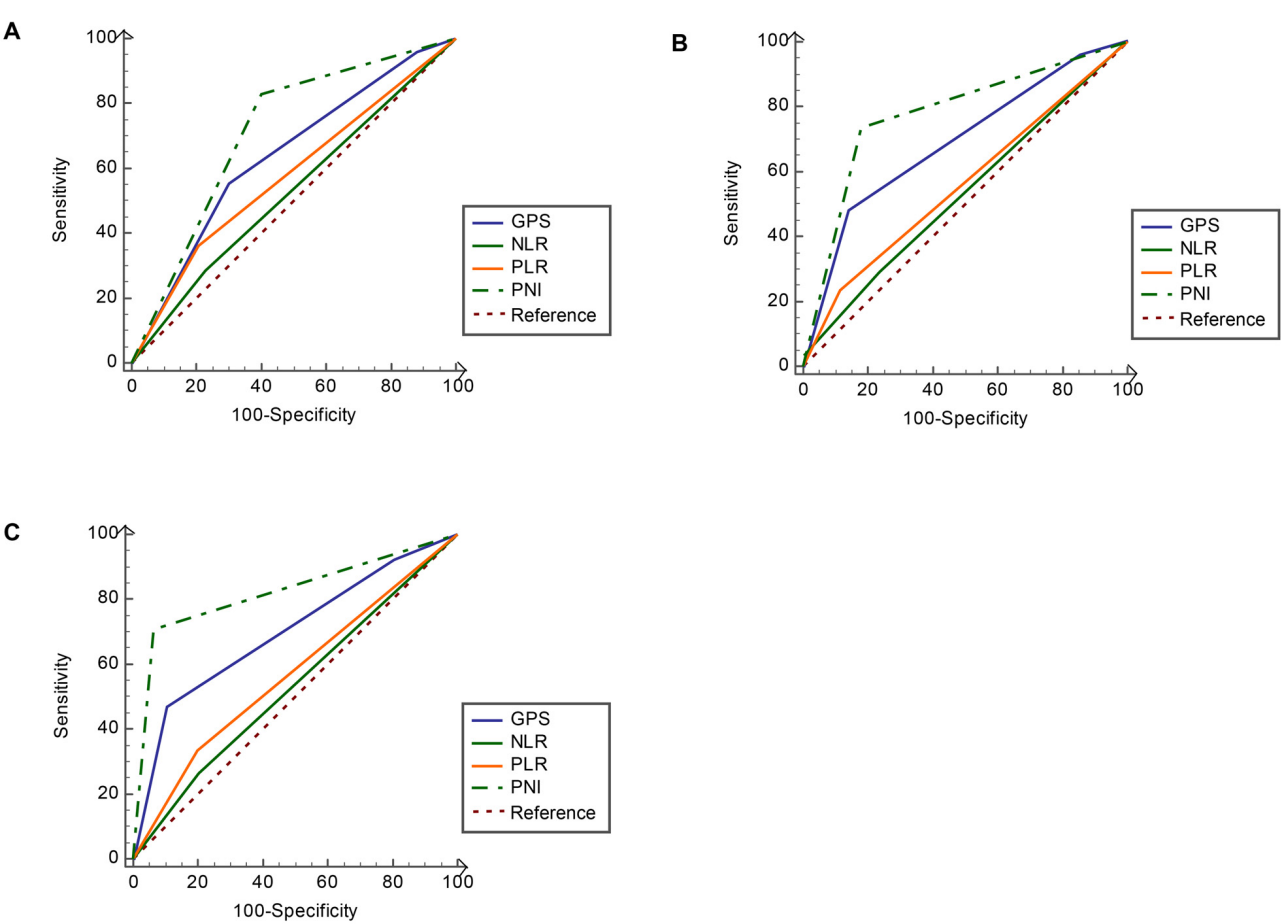
Comparisons of the area under the receiver operating curve (AUC) for predicting distant metastasis free survival (DMFS) by PNI, mGPS, PLR and NLR. (A) At 1-year (AUC = 0.780, 0.705, 0.673 and 0.572, respectively), (B) 3-year (AUC = 0.793, 0.711, 0.653 and 0.542, respectively) and (C) 5-year (AUC = 0.812, 0.715, 0.642 and 0.530, respectively).NLR, neutrophil to lymphocyte ratio; PLR, the platelet to lymphocyte ratio; mGPS, the modified Glasgow Prognostic Score; PNI, prognostic nutritional index.

Several inflammation-based scores were also confirmed to be associated with DMFS of NPC patients (S1 Fig). However, the PNI consistently show a higher AUC value at 1-year (0.780), 3-year (0.793) and 5-year (0.812) of follow-up in comparison with other inflammation-based prognostic scores (Fig 4)

## Discussion

To our knowledge, this is the first study to assess the prognostic value of PNI in patients with NPC treated with definitive radiotherapy. PNI was identified as a strong prognostic factor for DMFS, even in subgroup analysis and after propensity score-matching. The prognostic ability of PNI was better than other inflammation-based prognostic scores. Therefore, PNI may represent a novel biomarker for individualized therapy in NPC patients.

The PNI, which combines serum albumin and the lymphocyte count has been demonstrated as a prognostic factor in several malignant tumors, including colorectal cancer, gastric cancer, malignant pleural mesothelioma, hepatocellular carcinoma, pancreatic cancer and small cell lung cancer [20–22, 26]. A low PNI implies a decrease in albumin and/or lymphocytes. Serum albumin is an important indicator of the host inflammatory response and nutritional status and has been shown to be related to the prognosis of cancer patients [17, 18]. Higher serum albumin may exert an anticancer effect by stabilizing the circulating levels of growth factors, anabolic hormones, inflammatory cytokines and oxidative stress markers, which are considered to play important roles in cancer progression[27]. For cancer patients, malnutrition and inflammatory responses could suppress the synthesis of albumin by hepatocytes[18] and alter the metabolic homeostasis in the tumor microenvironment[28]. Therefore, lower albumin might indicate impaired immunonutrional status. Another aspect of PNI, the absolute lymphocyte count has been assumed as a critical participant in preventing cancer by initiating cytotoxic immune response[29]. Lymphocytopenia is also reported to be associated with disease severity, poorer prognosis and decreased chemotherapeutic efficacy in cancer patients[30]. Taken together, this existing evidence indicates that malnutrition and lymphocytopenia may serve as indicators of chronically impaired immune system, and may collectively promote tumor development and progression and lead to poorer prognosis.

In this study, univariate analysis showed that higher PNI was significantly associated with better DMFS in non-metastatic NPC. Additionally, PNI was significantly associated with inflammation-based prognostic factors, including WBC, HGB and CRP, suggesting that the PNI may collectively represent the prognostic value of all of these indexes, thus raising the issue of a possible interference on the results. Thus, we further used the propensity score-matched analysis to avoid this potential confounding bias. Multivariate analysis demonstrated that the PNI remains independently associated with DMFS before and after propensity score matching. Moreover, subgroup analyses showed the consistency of the prognostic value of PNI for DMFS. Therefore, this study indicates that chronic systemic inflammatory response and malnutrition, as indicated by low PNI, is associated with poorer survival in NPC.

Actually, some other circulating biomarkers, such as the higher platelet count, CRP (C-Reactive protein) can also serve as poorer prognostic indicators for decreased NPC patients survival [31, 32]. Additionally, in previous studies, several immune/nutrition-based prognostic scores have been proposed to predict the prognosis of NPC patients, including NLR, PLR and mGPS (S1 Table). These and our results strongly indicate that chronic inflammation might represent an unfavorable situation for cancer patients. Interestingly, in our study, we found that the PNI had better discriminatory ability for predicting the 1-year, 3-year and 5-year DMFS than other inflammatory scores. All in all, these results show that PNI may represent a novel and promising inflammation-based prognostic score for NPC patients.

Radiotherapy alone has been the first curative treatment of NPC and the concomitant chemoradiotherapy appears to be new treatment modality for locoregionally advanced NPC [33]. During treatment, patients may suffer from many acute and late-onset complications such as mucositis, dysphagia, nausea, and vomiting during therapy and these symptoms could lead to dehydration, undernutrition and impaired immune functions[34]. Thus, it is important that we provide patients with accurate information about the magnitude of benefit and the balance against excessive chemoradiotherapy toxicities. This study demonstrates that PNI, a widely available and inexpensive biomarker was strongly associated with DMFS in NPC patients after definitive radiotherapy. Therefore, there is clear justification for routine assessment of the PNI in patients with non-metastatic NPC during and after treatment to predict risk of distant metastasis, modulation of the immunonutrional status of patients and apply individualized adjuvant treatment and/or follow-up strategies. Moreover, though not investigated in the present study, dynamic monitoring of PNI beyond baseline in NPC patients treated with radiotherapy of chemoradiotherapy might provide interesting information about the associations between PNI and treatment efficacy, prognosis as well as toxicities.

Currently, there are still 10–20% of non-metastatic NPC patients who are understaged at diagnosis because distant micro-metastases are undetectable by the conventional staging workup, CT or MRI [35]. There remains a staggering heterogeneity of clinical outcomes for patients with equivalent TNM classifications and watchful waiting could result in under-treatment of approximately 20%-40% patients with occult metastases [36]. It is now clear that host immune system and nutritional status also influence the treatment outcomes of cancer patients and may also be related to occult metastases. Therefore, applying additional predicting indexes that involve host functionality is of significance. In this study, we found that lower PNI was associated with higher lymph node metastasis. However, we also found that in lymph node positive patients, PNI could stratify patients into high and low risk of distant metastasis, suggesting the PNI may serve as a useful marker to more accurately prognosticate the survival outcome of patients with NPC especially those with lymph node metastases.

Our results also raise the issue of possible treatment-interference based on the PNI, i.e. adjuvant immunonutritional therapy. Intriguingly, non-steroidal anti-inflammatory (NSAI) agents have been reported to decrease the risk of mortality of several cancer types [37, 38]. Modulation of the immune system has become a fascinating strategy to improve the treatment outcome of cancer patients. Whether there is clinical benefit of adding NSAI agents to NPC patients deserves further investigation through clinical trials. Additionally, the survival rates of patients with low PNI values may potentially benefit from nutritional therapy, such as the administration of branched-chain amino acid-enriched nutritional support [39].

This study has some limitations. Firstly, the study was conducted retrospectively and selection bias may exist. However, we included a relatively large training cohort to assess the independent prognostic value of PNI for DMFS by adjusting for group effects and confounders via propensity score-matched analysis and robustly externally validated the prognostic value of the PNI in the validation cohort. The results consistently demonstrated the significant prognostic value of the PNI for DMFS. Of course, additional validation of the PNI is necessary in prospective datasets.

In summary, this study suggests that the prognostic value of the PNI, a continuous variable, may help to stratify patient outcomes more accurately. Measurement of the PNI during routine pretreatment assessments may help to refine current staging methods and treatment allocation, and may serve as a practical tool for individualized prognostication of DMFS to enable tailored post-treatment follow-up and/or adjuvant therapy and improve survival outcomes in patients with NPC.

## Supporting Information

S1 Fig Kaplan-Meier curves of different PNI NLR, PLR, mGPS for distant metastasis-free survival (DMFS) in the training cohort before matching (A, B, C, D); NLR, neutrophil to lymphocyte ratio; PLR, the platelet to lymphocyte ratio; mGPS, the modified Glasgow Prognostic Score; PNI, prognostic nutritional index. (PDF)

S1 Table Characteristics of studies regarding inflammation-based prognostic scoring systems for the prediction of survival in the NPC patients. (DOCX)

## Abbreviations

PNI: Prognostic nutritional index
DMFS: Distant metastasis-free survival
NPC: Nasopharyngeal carcinoma
AUC: Receiver operating characteristics
EBV: Epstein-Barr virus
ALB: Albumin
ALT: Alanine transaminase
AST: Aspartate transaminase
LDH: Lactate dehydrogenase
ALP: Alkaline phosphatase
WBC: White blood cell
HGB: Hemoglobin
CRP: C-reactive protein
MRI: Magnetic resonance imaging
NLR: Neutrophil to lymphocyte ratio
PLR: Platelet to lymphocyte ratio
mGPS: modified Glasgow Prognostic Score
HRs: Hazards ratios
PSM: Propensity score-match analysis
