# Supplementary material for: Low Prognostic Nutritional Index (PNI) Predicts Unfavorable Distant Metastasis-Free Survival in Nasopharyngeal Carcinoma: A Propensity Score-Matched Analysis
Source: PLoS One. 2016 Jul 11;11(7):e0158853. doi: 10.1371/journal.pone.0158853 (PMC4939954; doi:10.1371/journal.pone.0158853)
Supplement: S1 Fig — (PDF) [file pone.0158853.s001.pdf]

Supplementary figure

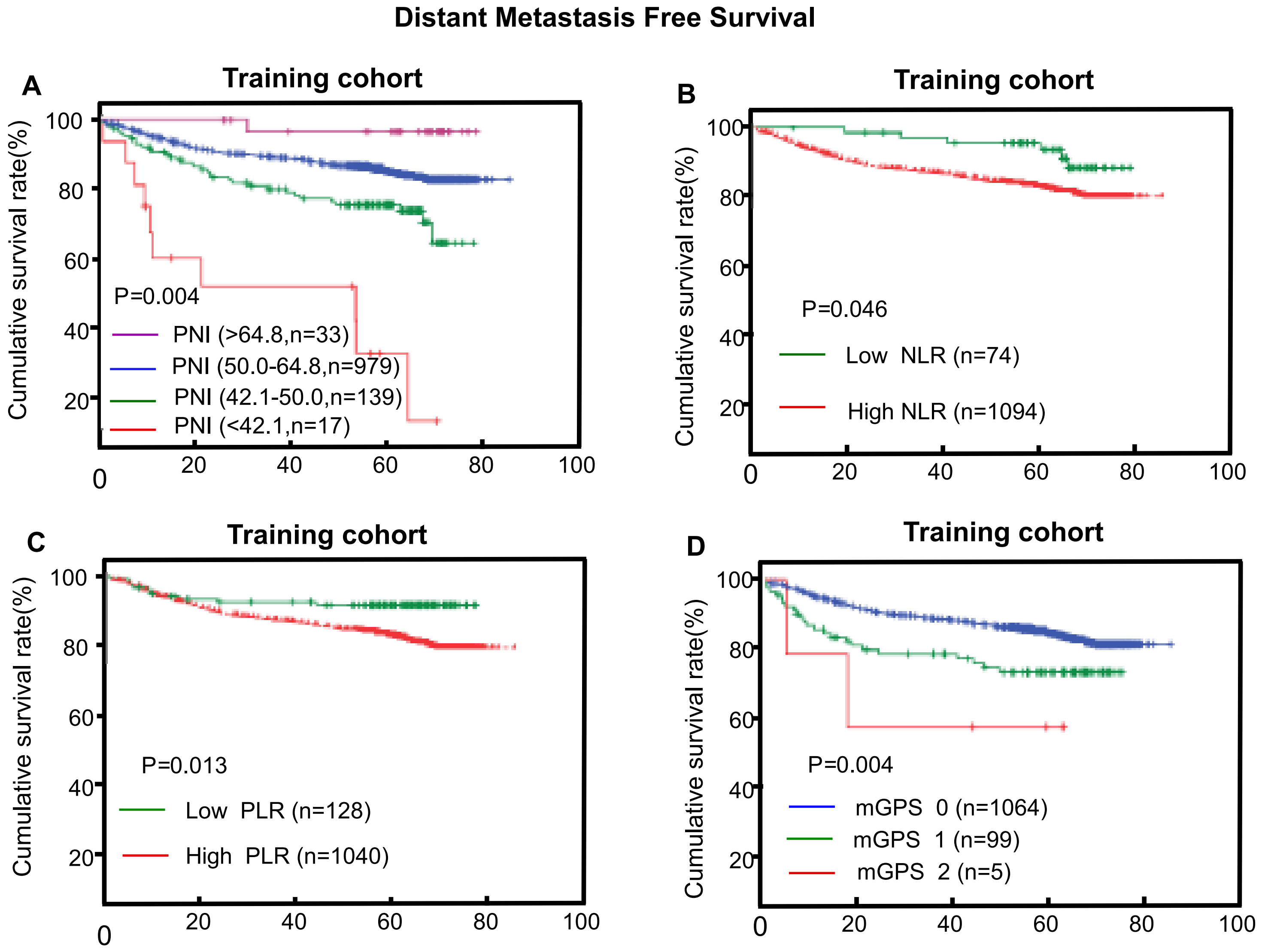

Supplementary Figure 1. Kaplan-Meier curves of different PNI NLR, PLR, mGPS for distant metastasis-free survival (DMFS) in the training cohort before matching
