# Supplementary material for: Low Prognostic Nutritional Index (PNI) Predicts Unfavorable Distant Metastasis-Free Survival in Nasopharyngeal Carcinoma: A Propensity Score-Matched Analysis
Source: PLoS One. 2016 Jul 11;11(7):e0158853. doi: 10.1371/journal.pone.0158853 (PMC4939954; doi:10.1371/journal.pone.0158853)
Supplement: S1 Table — (DOCX) [file pone.0158853.s002.docx]

**Supplementary table 1. Characteristics of studies regarding inflammation-based prognostic scoring systems for the prediction of survival in the NPC patients**

| **Model** | **Cut-off** | **Study** | **Region** | **Sample size (n)** | **HR(P-value)** | | | **Stage** |
| --- | --- | --- | --- | --- | --- | --- | --- | --- |
|  |  |  |  |  | **DMFS** | **OS** | **PFS** |  |
| **NLR** | 3.73 | Xin An[^1^](#_ENREF_1) | China | 381 | 2.37(0.002) |  |  | I-IVA |
| **NLR** | 2.74, 1.54 | Jian-Rong He[^2^](#_ENREF_2) | China | 1533 |  | 1.57(0.09)^*^ | 1.68(0.007) ^*^ | I-IVA |
| **NLR** | 4.76, 2.64 | Ying Jin[^3^](#_ENREF_3) | China | 229 |  | 1.906(0.001) ^*^ |  | IVB |
| **NLR** | 2.7 | Wei Sun[^4^](#_ENREF_4) | China | 251 |  |  | 2.01(0.005) | I-IVA |
| **PLR** | 167.2 | Wei Sun[^4^](#_ENREF_4) | China | 251 |  |  | 2.64(0.011) | I-IVA |
| **PLR** | 153.64 | Rou Jiang[^5^](#_ENREF_5) | China | 1261 | 1.58(0.009) | 1.87(0.001) |  | I-IVA |
| **mGPS** | 0/1/2 | Cui Chen[^6^](#_ENREF_6) | China | 211 |  | 2.520(<0.001) |  | IVB |

Abbreviations: NLR, the neutrophil to lymphocyte ratio; PLR, the platelet to lymphocyte ratio; mGPS, modified Glasgow Prognostic Score; HR, hazards ratio; DMFS, distant metastasis free survival; OS, overall survival; PFS, progression-free survival.

^*^Representing highest group vs. lowest group.

**Reference**

1. An X, Ding PR, Wang FH, Jiang WQ, Li YH. Elevated neutrophil to lymphocyte ratio predicts poor prognosis in nasopharyngeal carcinoma. Tumour biology : the journal of the International Society for Oncodevelopmental Biology and Medicine. Apr 2011;32(2):317-324.

2. He JR, Shen GP, Ren ZF, et al. Pretreatment levels of peripheral neutrophils and lymphocytes as independent prognostic factors in patients with nasopharyngeal carcinoma. Head & neck. Dec 2012;34(12):1769-1776.

3. Jin Y, Ye X, He C, Zhang B, Zhang Y. Pretreatment neutrophil-to-lymphocyte ratio as predictor of survival for patients with metastatic nasopharyngeal carcinoma. Head & neck. Jan 2015;37(1):69-75.

4. Sun W, Zhang L, Luo M, et al. Pretreatment hematologic markers as prognostic factors in patients with nasopharyngeal carcinoma: Neutrophil-lymphocyte ratio and platelet-lymphocyte ratio. Head & neck. Sep 11 2015.

5. Jiang R, Zou X, Hu W, et al. The elevated pretreatment platelet-to-lymphocyte ratio predicts poor outcome in nasopharyngeal carcinoma patients. Tumour biology : the journal of the International Society for Oncodevelopmental Biology and Medicine. Sep 2015;36(10):7775-7787.

6. Chen C, Sun P, Dai QS, Weng HW, Li HP, Ye S. The Glasgow Prognostic Score predicts poor survival in cisplatin-based treated patients with metastatic nasopharyngeal carcinoma. PloS one. 2014;9(11):e112581.
